# A National survey describing the quality of care in Paediatric Emergency Departments of Tertiary Hospitals in Nigeria

**DOI:** 10.4314/ahs.v24i2.35

**Published:** 2024-06

**Authors:** Callistus OA Enyuma, Abdullah E Laher, Muhammed Moolla, Feroza Motara, Gbenga Olorunfemi

**Affiliations:** 1 Department of Emergency Medicine, Faculty of Health Sciences, University of the Witwatersrand, Johannesburg, South Africa; 2 Department of Paediatrics, Faculty of Medicine, University of Calabar, Calabar, Nigeria; 3 Children Emergency Unit, Department of Paediatrics, University of Calabar Teaching Hospital, Calabar, Nigeria; 4 Division of Epidemiology and Biostatistics, School of Public Health, University of Witwatersrand, Johannesburg, South Africa

**Keywords:** Paediatrics, Emergency Department, Nigeria, quality of care, National survey

## Abstract

**Introduction:**

The outcome of paediatric emergency care is essential to the attainment of child-targeted sustainable development goals. We assessed the quality of paediatric emergency care among 34 tertiary Paediatric Emergency Departments (PED) in Nigeria.

**Methods:**

We conducted a cross-sectional process audit of recruited 34 PEDs in Nigeria. A paper questionnaire developed from the validated AAP/IFEM Guidelines for Care of Children in the ED was used to collect information on the PED settings, the processes of care and measurable patient outcome. Association between the regions, hospital volume category and other institutional attributes was conducted using chi-square,

**Results:**

The median (IQR) of paediatric visits and admissions to PEDs were 187.5 (120 - 300) and 107.5 (67 - 131) respectively. Over two-thirds (73.6 %,) of the PEDs had no set target Time-To-Physician consultation and the median (IQR) Length-of-Hospital Stay was 48 (0-72) hours.

The majority of centres (90%) had patient safety tools but point-of-care-diagnostics (POCDs) were grossly deficient (23.5%). The mean protocol utilization score was 8.7 out of a maximum score of 34.

The national crude death rate was 33.8 per 1000 children and there was no statistically significant relationship between the crude death rate and volume of hospital visits, (p-value=0.45) or geopolitical zones (p-value = 0.68).

**Conclusion:**

There was nationwide poor protocol utilization and non-availability of POCDs coupled with a high mortality rate at the PEDs. Development and utilization of locally relevant protocols and improvement in the availability of POCDs are essential.

## Introduction

The provision of quality healthcare to every ill child in healthcare facilities is desirable and necessary to achieve a favourable outcome^1^. Studies evaluating the Quality of care (QOC) usually investigate the settings of care, the processes of care, inclusive of all interactions between personnel and patients (throughput), and the outcomes of the care^2^. There is difficulty in defining QOC because of the various spectrum of inequalities in medical care between resource-rich countries with organised healthcare systems and resource-poor countries without access to basic emergency care^2^. Thus, QOC is relative and the same standards cannot be utilised across the board. This scenario also explains the global variation in phases of development and access to paediatric emergency medicine (PEM) practice^3^. Nonetheless, there are baseline standards of QOC that health facilities and personnel should aim to surpass irrespective of the region.

The processes of care are easily assessed, modified or adapted with measurable performance indicators. On the other hand, the outcome aspect of QOC may be patient-dependent and subjective and therefore difficult to measure. However, the patient mortality rate is a measurable component of QOC that is usually studied^2^,^4^. Data are routinely collected on the volume of paediatric visits, admission morbidity, and 30-day mortality as well as re-admission rates in the Paediatric Emergency Department (PED) of high-income countries to improve the quality of service offered. A similar rigorous process audit that involves evaluation of QOC at PEDs is rare in most low and middle income countries (LMIC) such as Nigeria^1^,^4^.

In Nigeria as well as most LMICs, access to services in a dedicated PED is limited as the facilities are few and only available in tertiary-level hospitals. Therefore, a greater number of very ill children requiring acute care are still being treated in the general emergency departments (ED) of primary and secondary-level facilities that are shared with adult patients^3^,^5^–^10^. Also, a high healthcare expenditure by parents/caregivers does not only limit patient access but also the affordability of services-for-pay that all impinge on the quality of services at the ED which happens to be the easiest route to basic healthcare delivery, often by self-referrals^11^. On the other hand, the concentration of PEDs and skilled personnel in urban areas widen the gap in the quality of care rendered to the different populations of the country^11^. These Nigerian PEDs while providing services to their clients may be faced with several drawbacks that may interfere with the quality of care. Such include prolonged patient time-to-physician, deficient supplies and support services, deficient child safety protocols, high bed occupancy and lean clinical staff-patient-friendly facilities. The aforementioned throughput concerns may vary internationally and also within regions.^12^–^14^

Studies have shown that health systems with in-built regular audits with the associated evaluation of QOC usually have improved clinical outcomes. Furthermore, evaluation of QOC can help to understand challenges in the health system and proffer safeguards to protect the client (patients). Patients' safety is also enhanced in health systems that are regularly audited^1^,^4^. Despite the importance of health system audits and regular evaluation of QOC to improve patient outcomes, there is currently scanty data on the quality of care at PEDs in Nigeria.

Therefore, this study aims to assess the QOC through measurable indicators such as settings of care, throughput performance indicators, processes of care of the patient, the support services available for care and outcomes of PED services across various tertiary healthcare facilities in Nigeria. This study hopes to provide information for policymakers towards improving the QOC at PEDs in Nigeria

## Materials and methods

This cross-sectional, questionnaire-based study involved 34 PED in tertiary healthcare facilities in Nigeria. The study aimed to select one PED in each of the 36 states of the country and the Federal Capital Territory (FCT). In 16 states and FCT where there was more than one PED per state, simple random sampling was used to select one PED. However, of the 37 selected tertiary centres, only 34 centres responded. Ethical clearance to conduct this research was obtained from the University of The Witwatersrand Human Research Ethics Committee (HREC-medical) (M 1700445), and Nigerian Federal Ministry of Health (NHREC/01/01/2007-21/05/2017). In addition, administrators of the participating hospitals also gave written permission and consent for the study except for three PEDs that did not give permission. Hospital and staff identifying information were removed and replaced with a unique pin to anonymise the data.

After informed consent was obtained, the self-administered paper-based questionnaires were given to the HOD and unit nursing manager at each participating hospital and retrieved upon completion.

**Information collected covered;** 1) the hospital settings, staff-targeted infrastructures and methods of payment for services, 2) the volume of paediatric visits and admissions into the short-stay ward, the morbidity and mortalities over a 30 days period before administering the questionnaire, 3) the number of daily shift duty, academic meetings, ward rounds, 4) set target time-to-physician (TTP), the patient maximum length of stay (LOS), 5) child safety measures/protocol, point-of-care (POC) diagnostics and other support services available.

### Data management

The PEDs were classified into three categories based on the volume of paediatric visits used in a study^15^. The categories were; low (<100 patients), medium (100-499 patients) and high (>500 patients).

Time-To-Physician (TTP) is defined as the patient wait time for the physician's initial assessment. Calculated from the time of patient arrival in the PED to their contact with the physician^16^.

The patient length of hospital stay (LOS) is defined as the length of time from patient admission to when a patient is properly disposed of from the unit^16^.

The mean protocol utilization score was calculated as an average of the number of PEDS that had 34 written protocols listed in the questionnaire.

The national crude mortality rate (NCMR), is the aggregate of all the deaths in all the PEDs over the 30 days prior to administering the questionnaire and was divided by the aggregate of paediatric visits and multiplied by 1000.^17^,^18^

### Data analysis

Data collected were entered into an Excel spreadsheet (Microsoft® Excel®) and then exported to STATA 14 (Stata Corp. 2015. College Station, TX: Stata Corp LP) for analysis.

Continuous variables such as the volume of patient visits and the number of admissions as well as the deaths in the last 30 days were described as means (±standard deviation) if normally distributed or median (interquartile range) if not normally distributed. Categorical variables (such as Time-to- physician and the maximum length of stay) were reported using frequencies and percentages. Chi-square and Fisher's exact were used to assess the relationship between categorical variables (such as geopolitical zone) and categorical outcomes (such as categories of hospitals based on the volume of visits). The level of significance was set at a 95% confidence interval (p<0.05).

## Results

### Settings of the studied paediatric Emergency Departments

Most (n=29/34, 85.3%) of the PEDs were located in urban cities and only 6 (17.7%) conducted safety drills in the last year before the study. Almost all of the facilities had doctors' (n=32/34, 94.12%) and nurses (n=31/34, 91.18%) call rooms. Also, about three-quarters of PEDs (n=25/34, 73.5%) had access to a library, and 20 (58.82%) had internet access. Furthermore, all the PEDs indicated that services are paid through out-of-pocket health payment (OPP) by caregivers (Supplementary file 1).

**Supplementary file 1:** Settings of the studied 34 Paediatric Emergency Departments

Parameter	Frequency (n=34)	Percentages (%)
**Setting**		
Urban	29	85.29
Rural	5	14.71
**Access road**		
Yes	34	100.00
No	0	0.00
**Fencing**		
Yes	33	97.06
No	1	2.94
**Parking space**		
Yes	33	97.06
No	1	2.94
**Safety drills**		
Yes	6	17.65
No	28	82.35
**Payment method**		
Cash	34	100
Cashless	0	0
**Doctors room**		
Yes	32	94.12
No	2	5.88
**Nurses room**		
Yes	31	91.18
No	3	8.82
**Lounge**		
Yes	24	70.59
No	10	29.41
**Doctors quarters**		
yes	22	64.71
no	12	35.29
**Library**		
yes	25	73.53
no	9	26.47
**Internet access**		
yes	20	58.82
No	14	41.18
**Paediatric conference room**		
Yes	22	64.71
No	12	35.29
**Multimedia**		
Yes	25	73.53
No	9	26.47
**Security personnel**		
Yes	33	97.06
No	1	2.94

### The processes of care in the paediatric emergency departments

#### The performance indicators in PED

Only a few PEDs (n=9/34, 26.5%) had set a benchmark for patients' time-to-physician (TTP) and ranged between 5 to 60 minutes with a Median (IQR) of 10 (10-30) minutes. Also, most of the PEDs (n=25/34, 73.5%) had a set maximum length-of-hospital. Stay (LOS) before disposal in their short-stay ward was from 1 – 5 days with a Median (IQR) of 48 (0-72) hours.

Almost one-third (n=10/34, 29.4%) of the PED facilities run 8- hourly shift duty, while close to half had once-a-day ward rounds (n=15/34, 44.1%).

Only about one-tenth of the PEDs 11.8% (n=4/34) had five departmental academic meetings per month (Table 1).

**Table 1:** The set performance indicators in the 34 Paediatric Emergency Departments

Parameters	PED Frequency n=34	Percentages (%)
Maximum stay (hours) Median(IQR) 48 (0-72) hours		
No set target	9	26.47
24 -48	16	47.06
>48	9	26.47
**Time to physician's review (minutes) Median (IQR) 10(10-30) minutes**		
No set target	25	73.53
5-10	6	17.65
10-30	2	5.88
30-60	1	2.94
**Number of daily ward rounds**		
1	15	44.12
2	14	41.18
3	4	11.76
4	1	2.94
**Number of shift daily**		
2	24	70.59
3	10	29.41
**Department academic meetings (DAM)**		
1	12	35.29
2	6	17.65
3	8	23.53
4	4	11.76
5	4	11.76
**Personnel attending the DAM**		
All staff	17	50.00
Doctors	6	17.65
Doctors/nurses	5	14.71
Doctors/students	6	17.65

IQR: Interquartile range

#### Patient safety tools, point of care diagnostics and support services

Patient safety tools such as Meter rule, weighing scale, clock, drip stands and suctioning devices were available nationally in >90% of PEDs but only about 15% of PEDs had a critical item like resuscitation algorithm (n=5/34, 14.7%). Point-of-care (POC) diagnostics was grossly deficient as only one (2.9%) PED had a POC blood testing machine and eight (23.5%) had mobile X-rays. However, 28 (82.4%) PEDs had functional side-room laboratories (Supplementary file 2).

**Supplementary file 2:** Patient safety tools and point of care diagnostics availability to paediatric emergency departments

Safety tools	Frequency (n=34)	Percent (%)
Clock	34	100.00
Drip stands		
Drip stands	33	97.06
Meter rule	34	100.00
Neck collar	15	44.12
Neck collar	5	14.71
Resuscitation algorithm	5	14.71
Resuscitation documentation record	5	14.71
Suctioning devices	33	97.06
weighing scale	34	100.00
**Point of care diagnostics**		
Mobile Ultrasound	33	97.06
Mobile X-ray	8	23.53
POC blood testing	1	2.94
Side laboratory	28	82.35

The mean protocol utilization score among the PEDs was 8.9 out of a maximum score of 34 points. Most notably, only 14.7% (n=5/34) of PED respondents reported having a protocol for disease management (Supplementary file 3).

**Supplementary file 3:** Availability of clinical protocol in the Paediatric Emergency Departments

Type of Protocol	Frequency (n=34)	Percentages (%)
Airway management	4	11.76
Analgesics	9	26.47
Bereavement	7	20.59
Burns	7	20.59
Child abuse	6	17.65
Consent form	17	50.00
Critical care	5	14.71
Disaster	3	8.82
Disease management	5	14.71
Do Not Resuscitate	1	2.94
Drugs	14	41.18
Ed death	8	23.53
Equipment	11	32.35
Immunization	15	44.12
Inter facility lab referral	7	20.59
Inter facility radiology	7	20.59
Inter facility transfer	6	17.65
Intra facility transfer	9	26.47
Mass casualty	7	20.59
Neonatal resuscitation	24	70.59
Occupational safety	5	14.71
Paediatric resuscitation	16	47.06
Pains score	9	26.47
Patient discharge	17	50.00
Patient education	8	23.53
Patient surge	4	11.76
Patients restraints	3	8.82
Personal Protective Equipment (PPE)	11	32.35
Post Exposure Prophylaxis	20	58.82
Psychiatric patient in ED	2	5.88
Sedation	7	20.59
Trauma	6	17.65
Triage	16	47.06
Vascular access	7	20.59

The support services available to the PED are described in Table 2. Almost all centres (97.1%, n=33/34) had ultrasonography and about half (n=19/34, 55.9%) had a Computed Tomography scans while 94.1% (n=32/34) of PEDs had functional in-site blood bank services.

**Table 2:** Support services available to the 34 Paediatric Emergency Department

Support services	Frequency (n=34)	Percentage (%)
**Imaging services readily available to PED**		
Analogue X-ray machine	28	82.35
Bronchoscopy	8	23.53
Computer tomography Scan	19	55.88
Digital X-ray machine	23	67.65
Fluoroscopy	6	17.65
Magnetic Resonance Imaging	8	23.53
Mobile x-ray machine	8	23.53
Ultrasound	33	97.06
**Laboratory services readily available to PED**		
Blood bank	32	94.12
Blood products	16	47.06
Catheterization laboratory	2	5.88
Chemical pathology	32	94.12
Haematology	33	97.06
Histopathology	32	94.12
Microbiology	34	100.00
Packed cells volume	20	58.82
Side room laboratory	28	82.35
Toxicology	2	5.88
**Referral plan**		
Burns and plastic unit	25	73.53
Cardiothoracic unit	17	50.00
Community doctor	16	47.06
Imaging referral	23	67.65
Intensive care unit	28	82.35
Inter facility referral	18	52.94
Laboratory referral	25	73.53
Paediatric surgery	32	94.12
Trauma	26	76.47
**Other support services**		
Ambulance	32	94.12
Cafeteria	28	82.35
Generator	33	97.06
Mortuary	33	97.06
Oxygen plant	25	73.53
Patient transport	34	100.00
Plumbing staff	32	94.12
Record staff	33	97.06
Security staff	34	100.00
Ward assistants	33	97.06
Works department	34	100.00

#### Patient characteristics and outcomes

The number of paediatric emergency visits, the numbers admitted and the number of death in each facility PEDs in the last 30 days prior to the study were configured by paediatric patient volume category. (Table 3). The number of paediatric emergency visits to the PEDs ranged from 20 – 1500 with a median (IQR) of 187.5 (120 - 300) while the median (IQR) number of admissions to the PEDs was 107.5 (67 - 131).

**Table 3:** Hospital volume category and the crude death rate compared among the 34 paediatric emergency departments

State (n=34)	Number of patients seen in preceding 30 days N= 8,610 (%)	Number of patients admitted in preceding 30 days N=3,957 (%)	Number of deaths in preceding 30 days N= 292 (%)	Crude death rate (hospital crude death rate/1,000)	National crude death rate (per thousand)	P-value (crude death rate by hospital cat)
**Low paediatric volume hospital (<100 visits/30days**				**Median(IQR) 72.17(48.44 – 87.17)**		
F	20 (0.23)	30 (0.76)	2 (0.68)	100.0		
G	64 (0.74)	64 (1.62)	3 (1.03)	46.9		
J	53 (0.62)	41 (1.04)	5 (1.71)	94.3		
P	80 (0.93)	40 (1.01)	4 (1.37)	50.0		
**Medium paediatric volume hospital(100-499 visits/30days)**				Median(IQR): 38.78 (25-75)		
A	120 (1.40)	16 (0.40)	0 (0.00)	0.0		
B	300 (3.50)	79 (2.00)	8 (2.74)	26.7		
C	110 (1.30)	106 (2.70)	7 (2.40)	63.6		
D	147 (1.71)	67 (1.70)	20 (6.85)	136.1		
E	120 (1.40)	120 (3.03)	10 (3.43)	83.3		
I	117 (1.36)	98 (2.48)	7 (2.40)	59.8		
K	320 (3.72)	320 (8.10)	6 (2.05)	18.6		
M	300 (3.50)	50 (1.26)	5 (1.71)	16.7		
N	108 (1.25)	50 (1.26)	6 (2.05)	55.6		
O	200 (2.32)	120 (3.03)	7 (2.40)	35.0		
Q	100 (1.16)	100 (2.53)	8 (2.74)	80.0	**33.9**	**0.45**
R	200 (2.32)	120 (3.03)	5 (1.71)	25.0		
T	259 (3.00)	109 (2.75)	17 (5.82)	65.6		
U	150 (1.74)	45 (1.14)	3 (1.03)	20.0		
V	200 (2.32)	153 (3.90)	15 (5.14)	75.0		
Y	175 (2.03)	123 (3.11)	10 (3.43)	57.1		
Z	131 (1.52)	131 (3.31)	13 (4.45)	99.3		
A2	430 (5.00)	96 (2.43)	4 (1.37)	9.3		
B2	200 (2.32)	120 (3.03)	5 (1.71)	25.0		
C2	223 (2.60)	200 (5.05)	7 (2.40)	31.4		
D2	120 (1.40)	75 (1.90)	10 (3.43)	83.3		
E2	138 (1.60)	101 (2.55)	5 (1.71)	36.2		
F2	200 (2.32)	150 (3.80)	6 (2.05)	30.0		
G2	426 (4.95)	167 (4.22)	16 (5.50)	37.6		
H2	250 (2.90)	130 (3.30)	10 (3.43)	40.0		
I2	140 (1.63)	110 (2.80)	13 (4.45)	92.6		
**High paediatric volume hospital(≥500visits/30days)**				Median(IQR) 14.33(9.03 – 26.52)		
H	500 (5.81)	200 (5.05)	6 (2.05)	12.0		
L	659 (7.65)	96 (2.43)	4 (1.37)	6.1		
S	1500 (17.42)	250 (6.32)	25 (8.60)	16.7		
X	550 (6.39)	280 (7.10)	20 (6.85)	36.4		

Alphabets and alpha numerals = annonymized 34 PEDs ; IQR:Interquartile range

The median (IQR) deaths across the PEDs was 7 (5 -10) patients and the national crude death rate was 33.9 per 1000 children. However, there was no statistically significant relationship between the hospital paediatric volume category and the crude death rate (p=0.45).

Table 4 shows that the South-South (60%) followed by North-Central (59.5) had the highest crude death rate while South-West had the lowest (23.7%). The hospital paediatric volume category (p=0.68) and the crude death rate (p=0.46) when compared among the six geopolitical zones were not significantly different.

**Table 4:** Hospital volume category and crude death rate compared among the 6 geopolitical zones of Nigeria

Geopolitical zones	Low paediatric volume hospital^$^	Medium paediatric volume hospital^$$^	High paediatric volume hospital^$$$^	P value)	Crude death rate	P value
North East	0 (0.0)	4 (15.4)	1 (25.0)	0.68	39.9 (±26.7)	0.46*
North Central	1 (25.0)	6 (23.1)	0 (0.0)		59.5 (±28.0)	
North West	0 (0.0)	5 (19.2)	1 (25.0)	χ^2^	51.7 (±31.9)	
South East	1 (25.0)	4 (15.4)	0 (0.0)	=7.50	52.1 (±52.2)	
South South	2 (50.0)	3 (11.5)	1 (25.0)		60.0 (±35.4)	
South West	0 (0.0)	4 (15.4)	1 (25.0)		23.7 (±10.9)	
Total	4	26	4			

Severe malaria (n= 32/34, 94.1%), diarrhoeal diseases (n=25/34, 73.5%) and pneumonia (n= 24/34, 70.6%) were the leading causes of admission (Table 5) while severe malaria (n= 32/34, 94.1%), sepsis (n=29/34, 85.3%) and CNS infections (n=21/34, 61.8%) were the leading causes of death (Table 6).

**Table 5:** Common causes of admissions in the 34 Paediatric Emergency Departments

Morbidities	Frequency (n=34)	Percentage (%)
Severe malaria	32.00	94.12
Diarrhoeal disease	25.00	73.53
Pneumonia	24.00	70.59
Acute severe asthma	16.00	47.06
Sickle cell anaemia complications	15.00	44.12
Sepsis	14.00	41.18
Central nervous system infections	12.00	35.29
Severe anaemia	10.00	29.41
Febrile convulsion	7.00	20.59
Severe acute malnutrition	4.00	11.76
Bronchiolitis	2.00	5.88
Hypoglycaemia	1.00	2.94
Post neonatal jaundice	1.00	2.94
Tonsillitis	1.00	2.94
Road traffic accidents	1.00	2.94
Snake bites	1.00	2.94

**Table 6:** Common causes of death in the 34 Paediatric Emergency Departments

Causes of death	Frequency (n=34)	Percentage (%)
Severe malaria	32.00	94.12
Sepsis	29.00	85.29
Central nervous infections	21.00	61.76
Pneumonias	19.00	55.88
Diarrhoeal disease	16.00	47.06
Severe anaemia	12,00	35.29
Severe acute malnutrition	8.00	23.53
Sickle cell anaemia complications	4.00	11.76
Malignancies	4.00	11.76
Respiratory failure	3.00	8.82
Post neonatal Jaundice	1.00	2.94
Road traffic accidents	1.00	2.94
Post neonatal Tetanus	1.00	2.94
Aspiration	1.00	2.94
Typhoid enteritis	1.00	2.94
Supraventricular Tachycardia	1.00	2.94

## Discussion

This study evaluated the national and subnational variations in settings, throughput performance indicators of care and outcomes in PEDs across Nigeria. It is hoped that results from our study will impact policymakers to position the PEDs in Nigeria for better service delivery and outcome.

### Settings of the paediatric emergency departments in Nigeria

Settings which encompass location (rural and urban) and facilities (infrastructural, equipment/supplies and person-friendly features) impact services in various ways and improved well-being and working-condition of healthcare personnel in rendering quality paediatric care cannot be over-emphasised^19^,^20^. The distribution of skilled manpower is globally skewed towards urban settings. In our study 85.3% (n=29/34) of the dedicated PEDs in Nigeria were located in tertiary hospitals in regional/state capitals while over 64% of her population reside in the rural areas^18^,^21^. Though this aligns with other studies conducted in the USA and Saudi Arabia, their situation differs because their EDs are equitably distributed and equipped to treat paediatric patients. Also, their centres in urban areas are more likely to have designated PED and not strictly in tertiary-level hospitals^22^–^24^. Furthermore, incentives to attract skilled paediatric emergency practitioners to the rural setting are lacking in most LMICs^25^. To mitigate this draw back the Federal and State Government's efforts should be geared towards establishing tertiary hospitals in all the senatorial districts of the country to bring tertiary paediatric care closer to the rural dwellers.

PED clinical staff in more than half of the studied facilities had access to internet connectivity, a departmental library, a conference room and a staff lounge. These facilities are expected to improve overall the quality of service delivered at these centres. However, only 22 (64.7%) centres were reported to have doctor's quarters. The availability of doctors' quarters for emergency staff in a country such as Nigeria is very central to improved quality of care as it may be difficult for doctors to get to the hospital promptly during off hours. Having doctors' quarters within the vicinity of the hospital also helps to obviate communication and security challenges that are common in the country. Unfortunately, we could not find studies to compare with.

Acute illnesses and childhood mortality do not only present both financial and psychological burdens to the parents, but also constraints to the attending clinical staff. In Nigeria, the paediatric mortality in tertiary hospitals PED is between 2-17.5% majorly due to late presentation, delayed interventions, financial constraints, unavailability of life-saving equipment and inadequate support services^26^,^27^,^36^,^28^–^35^. Although some of these morbidities can be correctly managed in most healthcare facilities, a critical percentage needs specialist care in a dedicated PED. Our study showed that all the emergency care services were largely fundedby parents' out-of-pocket-payment (OPP) despite the existence of the National Health Insurance Scheme (NHIS) in the country for thirteen years. The finding is similar to a report from a study in Pakistan that showed that 70% of their population access healthcare through private hospitals at fee-for-service^9^. According to a WHO report, 63.3% of Nigeria's total health expenditure was from private spending, of which 95.4% was OPP^37^,^38^. This figure is staggering when compared to the OPP of 6.6% in South Africa, 36.1% in Malaysia, and 16.8% in Turkey38,39. The NHIS at present covers less than 5% of the over 180 million Nigerians, basically Federal civil servants and a few organized private sectors^40^,^41^. This penetrance is discouraging when compared to the coverage rate of 80% in South Africa, 68.1% in Ethiopia, 48% in Kenya and 32.8% in Ghana^42^-^45^.

The implication for the uninsured is that in critical illness when time and availability of resources matter, patient families are made to pay for all services before the institution of treatment. Resorting to limited personal funds (OPP) impacts parent's ability to present the child early to the PED, procure the prescribed medicine and supplies, and also pay for the necessary investigations and procedures as well as boarding^40^. This will negatively impact the timing and effectiveness of intervention(s) on the child, not being able to or delayed procurement of the prescribed medicine, and paying for critical investigations to guide patient treatment. All these throughput challenges occasioned by OPP can indirectly affect the outcomes of care which will include patients leaving without being seen by the doctor (PLWBS), left against medical advice (LAMA), discharge against medical advice (DAMA), case fatality or development of other morbidities^40^,^41^. Thus, there is a need for a conscious effort by the government to guarantee the delivery of timely quality emergency care to the ill and or injured Nigerian child.

### The processes of care in the studied Paediatric Emergency Departments

#### The performance indicators in PED

About one-third (14.7%) of the PEDs targets 10 minutes as set TTP for all Paediatric Emergency visits. Achieving such a time frame will reduce the time to definitive clinical interventions thereby improving morbidity and mortality. This target TPP among Nigerian PEDs that set a benchmark is the lowest when viewed globally as most TTP ranges between 30 min (15 min of triage and 15 min wait time) in a Saudi Arabian study^46^ while other studies^46^–^48^ reported 120 minutes. This shorter TPP can be explained by the non-application of a formal patient triaging tool that assigns patient's to a category. This arbitrary sorting shortened the TPP and probably the time of intervention by the attending physician by short-circuiting the process of care and may prolong the time spent with the physician (TWP), an aspect not studied by us. Prolonging TWP may contribute to increased physician workload and ED crowding which compromise the quality of care. For PEDs in Nigeria to offer a globally comparable quality of care to their clients, we recommend that an evidence-based and realistic TPP target should be established by each ED.

In this study, a good number of facilities (n=22, 64.7%) set 48-72 hours as the maximum LOS in their short-stay-ward. Studies showed that a target time of 4-8 hours is used as maximum LOS in most developed countries^49^–^51^. The set LOS benchmarked in our study was not uniformly met as we recorded a LOS range of 1-5 days with a median of 48 hours. The patient/caregivers perception of the outcome of care, a key aspect of quality of care, will be impacted by their prolonged stay. The extended LOS could be attributable to factors such as patient acuity, no bed space in the main ward, PED discharge processes and bottlenecks in other parts of the hospital.

In this study, a few (29.4%) of the PEDs conformed to the recommended eight hourly shift while the majority still operate the traditional two shifts of 8 am to 4 pm and night call duty from 4 pm to 8 am. The reason could be due to ED manpower shortages as three daily shifts will require an additional number of skilled staff. The Saudi Arabian study centre^46^ ran a three-shift duty as prevalent in most developed countries while the Taiwan study^48^ ran two-shift-duty days as was observed in most of our study centres (70.6%). Prolonged shifts have been shown to predispose staff to burnout syndrome and may compromise the quality of care rendered while shorter 8-hour shifts engender improved patient care and satisfaction as well as ensure the well-being of clinical staff^52^.

This study showed that the majority (n=15/34, 44.1%) of PEDs had once a day ward round. It has been documented that having more than one round a day improves the quality of care, limits unnecessary investigations, reduces patient LOS and mortality coupled with better patient satisfaction^51^. More (n=12/34, 35.3%) had one departmental academic meeting (DAM) per month with 50% (n=17) of facilities having all the PED staff in attendance. DAM is critical to patient care being a clinical audit where knowledge is exchanged, areas for re-enforcement or improvement are identified and unit policies are developed^53^. Therefore it is imperative that each facility must consciously benchmark TTP and LOS, adopt the 8hourly shift duty, frequent ward rounds and clinical audit as target indicators for Quality Improvement of PEDs services.

#### Patient safety tools, point of care diagnostics and support services

Patient safety is also part of quality care and any failure in safety measures or to have a plan exposes the child patient to avoidable harm and negatively impacts outcomes. Although the clinical staffs were aware of the existence of standard protocols for various management issues and scenarios in the PED, there was a general deficiency in the availability of written protocols for patient treatment, procedures, and safety. Only three (8.8%) facilities had the protocol for a disaster, four (11.8%) for patient surges and burns while seven (20.6%) facilities had an airway management protocol. Protocols ensure standards are adhered to, reduces ED crowding, prevent unnecessary boarding, saves costs and enhance patient satisfaction^54^.

In this study, only 6 (17.7%) of the facilities conducted safety drills. Institutions may have contingency plans to respond in an emergency, however, gaps can only be addressed when identified by simulations^55^. This is important because hospitals have peculiar characteristics that make it vulnerable such as; the presence of piped combustible gases or bedside gas cylinders, multi-layered hospital buildings, and it is the first port of call in disaster or emergencies. Hospitals also have a mixed population of non-ambulant patients that may need assistance and planned evacuation in an emergency^21^,^55^–^57^ Emergency practitioners should be conversant with both hospital protocols as well as that of national where they exist.

Point-Of-Care diagnostics capability was poor among the recruited PEDs, although a high proportion (82.4%) of the PEDs have a side-room laboratory providing haemoglobin estimation, blood glucose estimation, microscopy for malaria parasite and urinalysis. The functionality of this side-room laboratory needs future study for evaluation. POCD has been documented to hasten patient triaging, reduce ED crowding and promote early patient disposal, hence positively impacting patient outcomes^58^.

The PEDs had good support from the intra-facility routine laboratory and imaging as well as blood bank services which are associated with prompt diagnosis and definitive treatment. However, these services are rendered only on the patient's payment of a prescribed fee. This could be a barrier to quality care however, we did not study the turnaround time for laboratory/imaging reports to aid timeous patient care. Less than a third of PED had referral plans for the acutely ill child while over two-thirds had access to other support services and oxygen plants. Similar to a report by an Indian study^59^, all the PED facilities had ambulance services that are mostly for inter-facility patient transport with the absence of EMS practitioners and do not undertake emergency pick up of patients. This not only delays the transportation of sick children using unconventional means to PEDs but patients are accompanied by a person who does not have resuscitation skills. This may potentially expose children to further harm and does not support the delivery of quality emergency services.

#### Patient characteristics and outcomes

This study only included patients that were triaged to be seen in the PED and excluded patients that might have been re-directed to paediatric out-patient clinics. The majority (76.5%) of PEDs across the country was within the medium volume (100-499 paediatric visits per month) category during the preceding 30 days of this study. This contrasts with a US study that reported a higher proportion of low volume category hospitals (<150 visits/month)^15^.

Our study's median number of patient visits was 187.5 per month, approximately six patients per day, which is slightly lower than the 300 patients per month reported from a study in Uyo with similar settings^60^, but much lower than a Pakistani single centre study that triaged a total of 1269 patients per month to the PED^61^. Since the above studies operate largely on a fee for services, the differences reported could be due to differences in the number of available alternative facilities, the referral system in place and the population density of the hospital location. Also, their study was conducted in secondary-level facilities while ours was in tertiary-level facilities where access is premised on referral.

Finding from this study, when compared to those from other developed countries, saw about the same numbers as the two studies that reported 10 to 14 patient visits per day in the USA^15^,^49^ but fewer than 122.6 per day in Taiwan^62^, and 204 patients per day in 12-bed Saudi Arabian ED^63^. This paediatric visit volume is higher than our study and may be due to differences in study methodology. Furthermore, the cost of healthcare could explain the difference as our PEDs offered out-of-pocket expenditure compared to medical aid-funded healthcare in their study. Seeing too few a patient per day may not present enough training exposure to physicians while seeing too many a day increases the chance of physician burnout and the chance of medical errors hence affecting quality of care delivered.

The median number of patients that were admitted into the short-stay ward was 107.5 per month (3.58 per day). This is lower than studies in Saudi Arabia^63^, Brazil^64^ and Pakistan^61^ that admitted 8, 13.7 and 19 patients per day respectively while the study in Taiwan^62^ reported an admission rate of 18% – 25%. The reason for the higher admission rate in these resource-rich settings and or some centres in our study may be that they saw higher acuity patients, or the triage system selected only those who met the criteria for ED visit and admission or their populations accessed the hospitals better. The lower rate in our study may be related to limitations likely due to out-of-pocket healthcare expenditure leading to a higher LAMA (left against medical advice) rate or because most ED visits are by self-referral as parents used ED as an easy route to prompt care. So these cold cases were missed out by the arbitrary triage system used by most studied PED but were eventually streamed to OPD by the attending physician. This is not different from a large study in seven LMICs where half of the recruited hospital emergency room did not have a triage tool nor defined methodology for the initial assessment of patients^65^. Furthermore, the difference could also be due to differences in admission criteria to the short-stay ward in other settings.

The patient-clinical staff ratio and boarding capacity could variably affect both the number of patient's seen and the number admitted per day. This study did not however look at the staff-patient ratio which was relatively low in our previous study^14^.

From this study, the five common causes of children admission in the 34 PEDs were; severe malaria, diarrhoeal diseases, pneumonia, acute severe asthma and sickle cell anaemia complications while severe malaria, sepsis CNS infections, pneumonia and diarrhoea diseases complications were the five most common causes of death. These findings are in keeping with other studies from similar settings^28^,^30^–^36^. Instructively, CNS infection, which was not among the common causes of admission, contributed significantly to mortality while complications from the diarrhoeal disease are still a menace despite concerted global efforts over the years. Deaths from these age-long endemic diseases still question the quality of care in the PED but confounding events prior to patient presentation need to be considered. This calls for conscientious, intercalated public health intervention adapted to the region to mitigate this ominous trend.

The national crude death rate of 33.8 per 1000 was generated from an aggregate of the various studied 34 PED death rates. This is quite high and calls for a review of the care processes and settings with a view to introduce interventions with measurable outcomes. Furthermore, there was no significant relationship between the hospital volume categories and their geopolitical zones. The same is observed between the crude death rate and hospital paediatric volume category and also between the crude death rate and the six zones. The existence of all the studied PED in Tertiary healthcare facility having relatively the same level of manpower, equipped alike, and patronized by patients with similar characteristics may have levelled out environmental influence on health indices across the zones.

## Limitations

The study did not study the patient-clinical staff ratio, the turnaround time of the laboratory investigations, blood products request and imaging services which is an important aspects of quality of services. Also, the period of the study may have also influenced the morbidity and mortality pattern.

## Conclusion

The study settings were suitable to deliver basic paediatric emergency care although restricted to tertiary centres skewed to capital cities with reasonable staff-friendly facilities and offering services for a fee. There was a nationwide lack of a unified set of service indicators, poor protocol utilization and non-availability of Point-Of-Care-Diagnostics. To reduce mortality, there is a need to extend Paediatric Emergency Department to other tiers of healthcare delivery system, to develop by consensus locally relevant protocols and benchmark target indicators to ensure paediatric patient safety. These are an essential part of the performance and quality improvement of services rendered by the nations' PEDs.

## Recommendations

1. Government should engage a public-private partnership approach, having educated the stakeholders, to bridge the rural-urban disparity by strengthening existing primary and secondary healthcare facilities to provide paediatric emergency services in rural areas
2. Out-of-pocket healthcare expenditure not only deepen caregivers further into poverty but negatively impacts paediatric patient care outcomes. Government should make a conscious commitment to review and roll out full NHIS coverage and to also implement the National Health Act which will deliver “at least” free emergency care services to all Nigerian children.
3. A national key-persons audit committee should be constituted by the Ministry of Health and relevant paediatric faculties to conduct a lean process improvement study of the PEDs, develop a setting-specific target benchmarks and key-performance indicators to guarantee the efficiency of the existing PED. Also, peer ranking methods could be used to ensure quality care delivery.
4. There was a general deficiency in the availability of written protocols for patient treatment, procedures, and safety among the PEDS. Setting-specific emergency paediatric care guidelines should be adopted and enforced to safeguard patient's needing care in PEDs.
5. A national crude death rate of 33.8 per 1000, with Central Nervous System infection and diarrhoeal disease contributing significantly to mortality despite concerted global efforts over the years, is disturbing. This call for a review of the national approach to immunization efforts and diarrhoea training and treatment unit's penetrance.
6. A complete absence of public Emergency Medical Services. The Ministry of Education and Health should, in conjunction with relevant agencies, draw up a curriculum for EMS personnel training that will naturally attract the establishment of public Emergency Medical Services in Nigeria.

## Figures and Tables

**Supplementary file 4:** STROBE Statement—Checklist of items that should be included in reports of cross-sectional studies

Item No		Recommendation
**Title and abstract ✓**	1	(*a*) Indicate the study's design with a commonly used term in the title or the abstract
		(*b*) Provide in the abstract an informative and balanced summary of what was done and what was found
**Introduction**		
Background/rationale ✓	2	Explain the scientific background and rationale for the investigation being reported
Objectives ✓	3	State specific objectives, including any prespecified hypotheses
**Methods**		
Study design ✓	4	Present key elements of study design early in the paper
Setting ✓	5	Describe the setting, locations, and relevant dates, including periods of recruitment, exposure, follow-up, and data collection
Participants ✓	6	(*a*) Give the eligibility criteria, and the sources and methods of selection of participants
Variables ✓	7	Clearly define all outcomes, exposures, predictors, potential confounders, and effect modifiers. Give diagnostic criteria, if applicable
Data sources/measurement ✓	8*	For each variable of interest, give sources of data and details of methods of assessment (measurement). Describe comparability of assessment methods if there is more than one group
Bias ✓	9	Describe any efforts to address potential sources of bias
Study size ✓	10	Explain how the study size was arrived at
Quantitative variables ✓	11	Explain how quantitative variables were handled in the analyses. If applicable, describe which groupings were chosen and why
Statistical methods ✓	12	(*a*) Describe all statistical methods, including those used to control for confounding
		(*b*) Describe any methods used to examine subgroups and interactions
		(*c*) Explain how missing data were addressed
		(*d*) If applicable, describe analytical methods taking account of sampling strategy
		(*e*) Describe any sensitivity analyses
**Results**		
Participants ✓	13*	(a) Report numbers of individuals at each stage of study—eg numbers potentially eligible, examined for eligibility, confirmed eligible, included in the study, completing follow-up, and analysed
		(b) Give reasons for non-participation at each stage
		(c) Consider use of a flow diagram
Descriptive data ✓	14*	(a) Give characteristics of study participants (eg demographic, clinical, social) and information on exposures and potential confounders
		(b) Indicate number of participants with missing data for each variable of interest
Outcome data	15*	Report numbers of outcome events or summary measures
Main results	16	(*a*) Give unadjusted estimates and, if applicable, confounder-adjusted estimates and their precision (eg, 95% confidence interval). Make clear which confounders were adjusted for and why they were included
		(*b*) Report category boundaries when continuous variables were categorized
		(*c*) If relevant, consider translating estimates of relative risk into absolute risk for a meaningful time period
Other analyses	17	Report other analyses done—eg analyses of subgroups and interactions, and sensitivity analyses
**Discussion**		
Key results ✓	18	Summarise key results with reference to study objectives
Limitations ✓	19	Discuss limitations of the study, taking into account sources of potential bias or imprecision. Discuss both direction and magnitude of any potential bias
Interpretation ✓	20	Give a cautious overall interpretation of results considering objectives, limitations, multiplicity of analyses, results from similar studies, and other relevant evidence
Generalisability ✓	21	Discuss the generalisability (external validity) of the study results
**Other information**		
Funding ✓	22	Give the source of funding and the role of the funders for the present study and, if applicable, for the original study on which the present article is based

## Data Availability

The datasets generated and/or analysed during the current study are available from [corresponding author].
